# Test-to-PrEP: An Egocentric Approach to Promoting HIV Discussions and Resource Sharing in PrEP Clients’ Social Networks

**DOI:** 10.1007/s10461-025-04635-9

**Published:** 2025-02-10

**Authors:** E. Rodriguez, A. L. Johnson, L. Craker, S. Butts, M. Kanamori, Susanne Doblecki-Lewis

**Affiliations:** 1 Division of Prevention Science and Community Health Sciences, Department of Public Health, Miami, USA; 2 Division of Infectious Diseases, Department of Medicine, University of Miami, Miller School of Medicine, 1475 12th Avenue, Miami, FL 33136 USA

**Keywords:** HIV testing, HIV self-test kit, Social network, PrEP, HIV prevention

## Abstract

Test-to-PrEP, a bundled HIV self-test (HIVST) and PrEP educational kit was distributed to the social networks (SN) of PrEP clients using social network strategies (SNS). PrEP clients (*n* = 100) distributed kits to their SN members (*n* = 415). Multilevel analysis assessed the relationship between demographic/network characteristics and likelihood of PrEP discussions. Higher network density (e.g., the level of linkage among network members) and daily contact were associated with having previously talked about PrEP. Networks that were denser (more ties/connections), more frequently in contact, and had a relationship type that assumes greater closeness (proximal relations) resulted in greater likelihood of having had PrEP conversations. This work demonstrates the feasibility of using SNS to increase routine HIV testing and PrEP knowledge. The Test-To-PrEP SNS strategy is a promising tool to increase HIV prevention and care access to all who may benefit.

**Supplementary Information:**

The online version contains supplementary material available at 10.1007/s10461-025-04635-9.

## Resumen

Test-to-PrEP, es un paquete que incluye una prueba de autodiagnóstico del VIH (HIVST) y un kit educativo sobre PrEP. Este paquete fue distribuido en las redes sociales (RS) de los clientes de PrEP utilizando estrategias de redes sociales (ERS). Los clientes de PrEP (*n* = 100) distribuyeron los paquetes a las personas de sus RS (*n* = 415). El análisis multinivel evaluó la relación entre las características demográficas de los usuarios de PrEP y de sus redes con la probabilidad de tener conversaciones sobre PrEP. Una mayor densidad de la red (por ejemplo, el nivel de conexión entre los miembros de la red) y el contacto diario estuvieron asociados con haber hablado previamente sobre PrEP. Las redes más densas (con más vínculos/conexiones), con contacto más frecuente y relaciones de mayor proximidad resultaron en una mayor probabilidad de haber tenido conversaciones sobre PrEP. Este trabajo demuestra la viabilidad de usar estrategias de SNS para aumentar las pruebas rutinarias de VIH y el conocimiento sobre PrEP. La estrategia SNS de Test-To-PrEP es una herramienta prometedora para ampliar la prevención del VIH y el acceso a la atención a todos aquellos que puedan beneficiarse.

**Supplementary Information:**

The online version contains supplementary material available at 10.1007/s10461-025-04635-9.

## Introduction

The United States (US) Ending the HIV Epidemic (EHE) initiative commits to reducing new cases of human immunodeficiency virus (HIV) and to decreasing disparities in treatment and prevention strategies [1]. Early diagnosis, prevention, and scaling effective initiatives in high-incidence cities are key components of achieving the EHE goals. To address the ongoing HIV epidemic two of the EHE pillars are *Diagnose* and *Prevent*, of which HIV testing and pre-exposure prophylaxis (PrEP) are key aspects. As a high-incidence region, Miami-Dade County (MDC) is an EHE priority jurisdiction; however, the MDC HIV epidemic continues despite the availability of PrEP as an effective biomedical prevention strategy [1]. The ongoing epidemic highlights the importance of implementation strategies to engage those who could benefit from HIV testing and PrEP. Social network strategies (SNS) have proven effective at engaging individuals in HIV programs focused on education, testing (in-office and at-home self-testing), prevention, and retention in care [2–4].

SNS are largely based on the Social Contagion Theory and the Theory of Diffusion of Innovations; which together explore how behaviors, attitudes, and information spread through and are adopted within networks [5]. As such, engaging individuals who are central in their networks (i.e., connected to many network members) allows for the engagement of additional network members and enhances recruitment. Further, these central individuals promote the dissemination of the kit, expanding the reach beyond those who directly receive the kit [4]. Using a community-based participatory research (CBPR) approach, we developed and implemented Test-to-PrEP, a kit containing an HIV self-test kit and information regarding PrEP services [4]. Additional information regarding the Test-to-PrEP kit, including the kit’s design, and CBPR approaches used are described elsewhere [4]. Our project sought to leverage SNS to engage PrEP clients’ social networks in HIV testing and PrEP for prevention, and was successful in extending reach through this approach [4]. In this report we will detail the social network components of this project, including the distribution of kits through social networks (SN), the egocentric networks of the kit distributors, and participants’ willingness to encourage and discuss PrEP with their network members.

## Methods

### Human Subjects Research

Prior to study activities being conducted, approval was obtained from the University of Miami Institutional Review Board. Written or electronic consent in English or Spanish was provided and obtained from all participants.

### Project Overview

PrEP clients (e.g., egos) were recruited as Test-to-PrEP kit distributors (*n* = 100) during their routine PrEP visit at the University of Miami Rapid Access Wellness and Mobile PrEP Clinics. PrEP clients were offered up to four kits for distribution. Recruitment occurred from November 2021 to March 2022. Upon enrollment, PrEP clients completed a demographic questionnaire and a social network egocentric interview (*n* = 100). The egocentric interview focused on identifying network members the PrEP clients intended on distributing the Test-to-PrEP kits to (e.g., their alters). Additional information regarding project protocol, data collection procedures, implementation outcomes, and participant demographic information are available elsewhere [4].

### Social Network Approaches

**Fig. 1:**
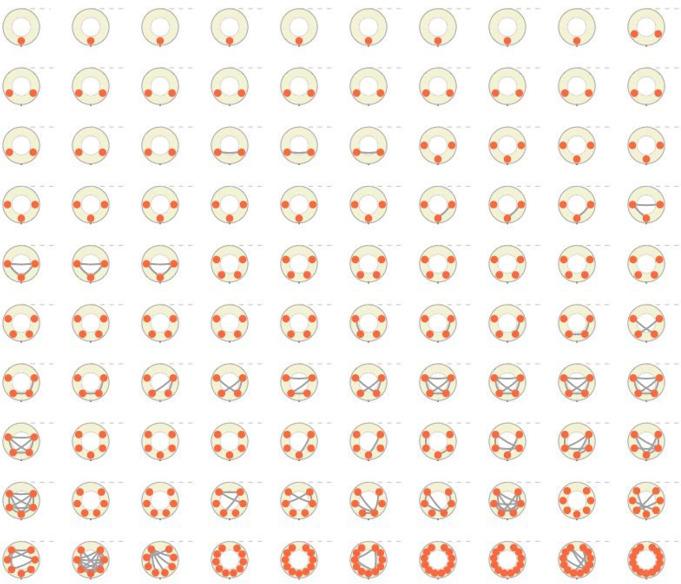
A visualization of egocentric networks provided by the existing PrEP clients. Each image represents the reported alters and the connections between alters

Egocentric interview approaches are a method in which one network member, the ego, reports on themselves and all other network members. Through egocentric approaches, information about the alters’ demographics and network characteristics (e.g., which alters know each other) are gained from the ego’s perspective. Using the egocentric approach egos were prompted to name up to 13 individuals who they believed could benefit from the kit, which resulted in a total of 415 alters. PrEP clients then described their relationship with the potential kit recipients, the frequency at which they discuss HIV and HIV prevention strategies including PrEP, and their willingness to discuss these topics in the future. These data were then used to create social network visualizations depicting the egos’ networks and the relationships between their alters (Fig. 1). A network was created for each ego, with a node (e.g., a circle) representing each reported alter; egos are not depicted in this visualization. Relationships between alters are depicted by ties, which are lines connecting nodes to each other. In this situation, ties represent if the nodes know each other, as reported by the ego during the egocentric interview.

### Ego-Level Characteristics

In part one of the baseline survey, egos provided their race, ethnicity, gender, sex assigned at birth, country of birth, the perceived gender of their sexual partner(s), whether they were a new or existing PrEP user at the time of enrollment, and the number of Test-to-PrEP kits they accepted.

### Ego-Alter Characteristics

In part two of the baseline survey, egos reported demographic information for up to 13 alters including their perceived age, gender, race, and ethnicity. Additionally, egos were asked to describe their relationship with the identified alter(s). Characteristics of ego-alter communications and interactions (in the past 6-months), perceived barriers, and facilitators to Test-to-PrEP kit distribution, and the strength of the connection between the ego and alter (e.g., closeness) were also captured. Egos were also asked about whether they had already discussed PrEP with the named alters (PrEP discussed), their likelihood to discuss PrEP in the future (PrEP talk), their likelihood to promote PrEP in the future (encourage PrEP use), and their likelihood to encourage the use of the HIVST kit in the future. Response options for having discussed PrEP with the named alters was binary in nature and this question served as the primary outcome for the multilevel logistic regression. Response options for likelihood to promote PrEP use in the future were ordinal with response options including "not likely at all", "not very likely", "a little likely", and "very likely". Response options for likelihood to encourage HIVST kit usage use in the future were ordinal with response options including “not likely at all”, “not very likely”, “a little likely”, and “very likely”. As the results were skewed, these ordinal options were transformed into binary variables; response options collapsed “not likely at all” and “not very likely” into one category (response option: No), and “a little likely” and “very likely” into the other (response option: Yes). Homophily, the social phenomenon in which people tend to cluster with those similar to them, was also evaluated [6]. Additionally, network density was calculated as the proportion of actual connections between alters to the total possible connections in the network, with values ranging between 0 and 1. A value of 1 indicates all possible connections exist (fully connected network), while 0 indicates no connections [6].

### Data Analysis

The outcomes of interest in the multilevel logistic regression analysis were whether egos had discussed PrEP with their alters (binary) and the likelihood to encourage HIV self-test kit use (binary). Demographic and other characteristics were summarized using frequencies and proportions for categorical variables and means and ranges for continuous variables. Descriptive statistics of egos’ characteristics and alters’ characteristics are available in Supplemental Materials.

Egocentric network data were analyzed using multilevel generalized linear models using logit link function for binary outcomes. The analysis of data recognized that alters were nested within participants’ social networks (egos), and a multilevel design was appropriate [7]. Level 1 included information from alters or ego-alter characteristics. Level 2 included information from participants (egos). The R computing environment was used for the creation of the network visualizations and for the completion of all analyses [8].

## Results

### Participant and Social Network Member (Alter) Characteristics

Participants reported a total of 415 network members (alters). Almost all participants reported sex assigned at birth as male (96%); the majority were not born in the United States (82%), identified as Hispanic/Latino (82%), and identified as White (61%). Participants were predominantly recruited at PrEP follow-up appointments and accepted a median of 3 HIV self-test kits. The mean network density was 0.25 with a range of 0–1. Alters were perceived largely as male (79.5%), Hispanic/Latino (75.9%), between 30 and 39 years old (47.5%), and were most commonly described as friends (63.1%). Alters were not described as main partners (84.1%) and relationships with alters were most commonly reported as moderately close (33.5%). Egos reported they had discussed PrEP with a total of 126 alters (30.4%), were likely to encourage PrEP use with 342 alters (82.4%) and were likely to encourage HIV self-testing with 369 alters (88.9%).

### Multilevel Analysis Results

Utilizing multilevel logistic regression we found higher network density and having contact every day to be associated with having previously talked about PrEP (OR: 1.01, *p*-value < 0.01; OR: 1.09, *p*-value = 0.04, respectively). Additionally, having a relationship characterized as neighbor was associated with a reduced likelihood of PrEP discussed (OR: 0.76, *p*-value = 0.03), when compared to more proximal relationships such as friends, family members, and sexual partners. Having some contact in the last 6-months (every month, every week, and every day) was positively associated with likelihood to encourage HIVST kit usage (OR: 3.98, *p*-value < 0.01; OR:5.19, *p*-value < 0.01; OR:5.21, *p*-value < 0.01, respectively). Additionally, perception of alter’s age as over 50 years was associated with a lower likelihood of encouraging HIV self-test kits.

### Network Visualization

Networks reported by each ego are depicted in Fig. 1. In this visualization, networks are arranged by the number of reported alters followed by the number of ties. This visualization demonstrates the variation in number of alters and network density across the egos.

## Discussion

We explored the SN of PrEP clients to understand the association between network structures, HIV self-test kit distribution, and PrEP conversations. Our findings highlight that having a network of individuals who are interconnected increases the odds of discussing PrEP. The density of a network related to the connectivity of one’s social network, thus suggesting that those who are more socially connected may be more likely to discuss HIV prevention through PrEP. When compared to more proximal relationships such as friends, family members, and sexual partners, being described solely as a neighbor was associated with a lower likelihood to have PrEP conversations, furthering the idea that a greater level of connectivity matters. Additionally, those who interacted more frequently were more likely to engage in conversations regarding PrEP and were more likely to encourage the use of HIV self-test kits among network members. Our findings are consistent with previous work that similarly found an association between PrEP-related conversations and contact frequency among the friendship networks of Latinx men who have sex with men [9]. Notably, the importance of contact frequency for both PrEP conversations and HIVST kit encouragement suggests that regardless of perceived closeness those who engage often can be successfully reached by participants who act as champions of HIV prevention.

Using a CBPR approach and SNS, this project and the Test-to-PrEP kit were developed to reach and engage MDC’s community members who are vulnerable to HIV but otherwise unengaged by traditional approaches. Our results demonstrate that SNS are able to engage these communities, reaching beyond communities frequently engaged by projects (e.g., men who have sex with men). Specifically, our egos described a willingness to distribute the kits to 76 alters they identified as female. Reaching women is a particularly exciting finding as the majority of interventions and PrEP outreach strategies focus on engaging men [10]. Additionally, Test-to-PrEP engages all sexual partners, not just “main partners”, further expanding the reach of the strategy.

Our work expands upon the literature that shows trusted organizations can work with network members to disseminate HIV testing and prevention information [11]. Egos in our project endorsed a willingness to disseminate kits and discuss PrEP with their network members. This willingness is noticeable as the majority of egos reported no previous PrEP discussions with their alters. Perhaps their willingness to engage stems from their ability to provide their alters with the Test-to-PrEP kit that contains information and testing options. Our findings indicate that this approach is a promising mechanism to increase HIV testing and PrEP awareness. Importantly, our approach aligns with literature which shows that individuals are motivated to engage in HIV prevention services when they learn about them from trusted sources, including close friends and sexual partners [12].

### Limitations

Due to the egocentric nature of this work, results may not be generalizable to other settings or other networks in which the social and cultural dynamics differ from MDC. The majority of our sample identified as Hispanic/Latino, which accurately reflects the demographics of MDC, but not other US settings. Furthermore, egocentric data only reflects the perceptions of those interviewed, the views of the alters may differ but are not captured in this method. Due to the anonymous nature of the kit recipients Qualtrics survey we are unable to determine if these respondents are the same alters described in the initial egocentric interview. Additionally, the use of skip logic in the survey resulted in a larger proportion of missing responses for ‘PrEP discussed,’ as this question was contingent on participants reporting kit distribution. This design, while efficient, may have biased our findings by excluding alters from less-engaged participants. Further, at the point of implementation, only oral PrEP had the approval of the Food and Drug Administration (FDA), thus participants only received information about oral PrEP. Additionally, participants only reported on their willingness to discuss oral PrEP which may differ from their willingness to discuss and encourage long-acting injectable PrEP (LAI-PrEP).

## Conclusion

This work demonstrates the feasibility of using SNS to increase routine HIV testing and PrEP knowledge. As such future work to measure the utility of SNS for HIVST and PrEP information through a randomized trial are planned. Additionally, future iterations of the Test-to-PrEP kit may include additional information about LAI-PrEP. This work identified important characteristics of social networks when used for distribution of HIVST and PrEP information. Networks that were denser (more ties/connections), more frequently in contact, and had a relationship type that assumes greater closeness resulted in greater likelihood of having had PrEP conversations. The Test-To-PrEP SNS strategy is a promising tool to increase HIV prevention and care access to all who may benefit.

## Electronic Supplementary Material

Below is the link to the electronic supplementary material.

Supplementary Material 1

Supplementary Material 2

## References

[1] Centers for Disease Control and Prevention, Ending the HIV Epidemic in the U.S. (EHE), Division of HIV Prevention, Editor. 2023: https://www.cdc.gov/endhiv/index.html

[2] King K, et al. Feasibility and acceptability of HIV Self-Test kit distribution through PrEP clients’ social and sexual networks to increase HIV Testing and PrEP Information. J Acquir Immune Defic Syndr. 2022;90(S1):S105–13. 10.1097/QAI.0000000000002970. 35703762 10.1097/QAI.0000000000002970 PMC9204857

[3] Kanamori M, et al. Progreso en Salud: findings from two adapted Social Network HIV Risk reduction interventions for Latina Seasonal Workers. Int J Environ Res Public Health. 2019;16(22):4530. 10.3390/ijerph16224530. 31731821 10.3390/ijerph16224530 PMC6888294

[4] Johnson AL, et al. Test-To-PrEP: assessing Reach and Adoption of a New Approach to increase HIV Testing and PrEP Knowledge using HIV Self-Test kit distribution through PrEP clients’ Social Networks. J Acquir Immune Defic Syndr. 2023;94(5):421–8. 10.1097/QAI.0000000000003294. 37949445 10.1097/QAI.0000000000003294 PMC10651164

[5] Valente TW, Vega Yon GG. Diffusion/Contagion Processes on Social Networks. Health Education & Behavior. 2020;47(2):235-248.10.1177/1090198120901497. 10.1177/1090198120901497 PMC13528246 32090655

[6] Yang S, Keller FB, Zheng L. Social Network Analysis: Methods and Examples. 2017: Thousand Oaks, California.10.4135/9781071802847.

[7] Perry B, Pescosolido B, Borgatti S. Egocentric Network Analysis: Foundations, Methods, and Models. 2018.10.1017/9781316443255

[8] Core Team R. R: a language and environment for statistical computing. Vienna, Austria: R Foundation for Statistical Computing; 2021.

[9] Shrader C-H et al. The Association between Social and spatial closeness with PrEP conversations among latino men who have sex with men. Jaids-journal of acquired immune deficiency syndromes, 2021. 88(4): pp. 366–75.10.1097/QAI.0000000000002777 10.1097/QAI.0000000000002777 PMC8556301 34342298

[10] HIV Prevention Research Synthesis Project, Compendium of Evidence-Based Interventions and Best Practices for HIV Prevention., Centers for Disease Control and Prevention, Editor. 2022: https://www.cdc.gov/hiv/research/interventionresearch/compendium/index.html

[11] Stojanovski K, et al. A systematic review of the Social Network Strategy to optimize HIV Testing in Key populations to end the epidemic in the United States. AIDS Behav. 2021;25(9):2680–98. 10.1007/s10461-021-03259-z. 33871730 10.1007/s10461-021-03259-z PMC8054132

[12] Walsh JL, et al. Sources of information about Pre-exposure Prophylaxis (PrEP) and associations with PrEP Stigma, intentions, Provider discussions, and use in the United States. J sex Res. 2023;60(5):728–40. 10.1080/00224499.2022.2110208. 36036718 10.1080/00224499.2022.2110208 PMC9971350

