# Supplementary material for: Test-to-PrEP: An Egocentric Approach to Promoting HIV Discussions and Resource Sharing in PrEP Clients’ Social Networks
Source: AIDS Behav. 2025 Feb 10;29(5):1663–8. doi: 10.1007/s10461-025-04635-9 (PMC12031874; doi:10.1007/s10461-025-04635-9)
Supplement: Supplementary file 1 — Supplementary Material 1 [file 10461_2025_4635_MOESM1_ESM.docx]

Table 1: Characteristics of the PrEP Clients (Egos) Who Distributed the Test-To-PrEP Kit to Their Social Networks.

| **Table 1: Ego Characteristics** | **Overall (N=100)** |
| --- | --- |
| **Born in the United States** |  |
| No | 82 (82.0%) |
| Yes | 18 (18.0%) |
| **Ethnicity** |  |
| Hispanic | 82 (82.0%) |
| Non-Hispanic | 18 (18.0%) |
| **Race** |  |
| American Indian or Pacific Islander | 1 (1.0%) |
| Asian | 1 (1.0%) |
| Black or African American | 9 (9.0%) |
| Multi-racial | 6 (6.0%) |
| Other | 22 (22.0%) |
| White | 61 (61.0%) |
| **Sex assigned at birth** |  |
| Female | 4 (4.0%) |
| Male | 96 (96.0%) |
| **Recruitment Type** |  |
| Network Member | 5 (5.0%) |
| PrEP Follow-Up | 74 (74.0%) |
| PrEP Initial | 21 (21.0%) |
| **Number of kits accepted** |  |
| Mean (SD) | 2.94 (1.16) |
| Median [Min, Max] | 3.00 [1.00, 4.00] |
