# Supplementary material for: Test-to-PrEP: An Egocentric Approach to Promoting HIV Discussions and Resource Sharing in PrEP Clients’ Social Networks
Source: AIDS Behav. 2025 Feb 10;29(5):1663–8. doi: 10.1007/s10461-025-04635-9 (PMC12031874; doi:10.1007/s10461-025-04635-9)
Supplement: Supplementary file 2 — Supplementary Material 2 [file 10461_2025_4635_MOESM2_ESM.docx]

Table 2: Characteristics of Social Network Members (Alter) Described by the PrEP Clients (Egos) as the Test-To-PrEP Kit Recipients.

| **Table 2: Alter Characteristics** | **Overall (N=415)** |  | **Alter Characteristics** | **Overall (N=415)** |
| --- | --- | --- | --- | --- |
| **Ethnicity** |  |  | **Member Age Range** |  |
| Hispanic | 315 (75.9%) |  | 18 to 29 years | 120 (28.9%) |
| Non-Hispanic | 96 (23.1%) |  | 30 to 39 years | 197 (47.5%) |
| Missing | 4 (1.0%) |  | 40 to 49 years | 65 (15.7%) |
| **Gender** |  |  | 50 years + | 29 (7.0%) |
| Male | 330 (79.5%) |  | Missing | 4 (1.0%) |
| Female | 75 (18.1%) |  | **Main Partner** |  |
| Trans spectrum M-to-F | 1 (0.2%) |  | Yes | 34 (8.2%) |
| Trans spectrum F-to-M | 2 (0.5%) |  | No | 349 (84.1%) |
| Missing | 7 (1.7%) |  | Missing | 32 (7.7%) |
| **Closeness** |  |  | **Contact Frequency** |  |
| Not close at all | 30 (7.2%) |  | Never | 39 (9.4%) |
| Slightly close | 62 (14.9%) |  | About every month | 159 (38.3%) |
| Moderately close | 139 (33.5%) |  | About every week | 154 (37.1%) |
| Very close | 109 (26.3%) |  | About every day | 62 (14.9%) |
| Extremely close | 69 (16.6%) |  | **Relationship** |  |
| Missing | 6 (1.4%) |  | Friend | 262 (63.1%) |
| **Encourage PrEP Use** |  |  | Sexual Partner | 64 (15.4%) |
| Not likely at all | 27 (6.5%) |  | Co-worker | 33 (8.0%) |
| Not very likely | 45 (10.8%) |  | Neighbor | 15 (3.6%) |
| A little likely | 93 (22.4%) |  | Family Member | 31 (7.5%) |
| Very likely | 249 (60.0%) |  | Missing | 10 (2.4%) |
| Missing | 1 (0.2%) |  | **PrEP Talk** |  |
| **Encourage Self-Testing** |  |  | Not likely at all | 160 (38.6%) |
| Not likely at all | 10 (2.4%) |  | Not very likely | 83 (20.0%) |
| Not very likely | 29 (7.0%) |  | A little likely | 75 (18.1%) |
| A little likely | 58 (14.0%) |  | Very likely | 87 (21.0%) |
| Very likely | 311 (74.9%) |  | Missing | 10 (2.4%) |
| Missing | 7 (1.7%) |  | **Self-Test Kit Talk** |  |
| **PrEP Discussed** |  |  | Not likely at all | 13 (3.1%) |
| Talked about PrEP | 126 (30.4%) |  | Not very likely | 18 (4.3%) |
| Have not talked about PrEP | 64 (15.4%) |  | A little likely | 68 (16.4%) |
| Missing | 225 (54.2%) |  | Very likely | 309 (74.5%) |
